# Mitochondrial determinants of mammalian longevity

**DOI:** 10.1098/rsob.170083

**Published:** 2017-10-25

**Authors:** Yasuhiro Kitazoe, Masami Hasegawa, Masashi Tanaka, Midori Futami, Junichiro Futami

**Affiliations:** 1 Center of Medical Information Science, Kochi Medical School, Nankoku, Kochi 783-8505, Japan; 2 Institute of Statistical Mathematics, Midori-cho 10-3, Tachikawa, Tokyo 190-8562, Japan; 3 Department of Genomics for Longevity and Health, Tokyo Metropolitan Institute of Gerontology, 35-2 Sakae-cho, Itabashi, Tokyo 173-0015, Japan; 4 Department of Biomedical Engineering, Faculty of Engineering, Okayama University of Science, 1-1 Ridaicho, Okayama 700-0005, Japan; 5 Department of Biotechnology, Graduate School of Natural Science and Technology, Okayama University, Okayama 700-8530, Japan

**Keywords:** ageing theory, mammalian longevity, metabolic rate, mitochondrial membrane proteins

## Abstract

Current ageing theories are far from satisfactory because of the many determinants involved in ageing. The well-known rate-of-living theory assumes that the product (lifetime energy expenditure, LEE) of maximum lifespan (MLS) and mass-specific basal metabolic rate (msBMR) is approximately constant. Although this theory provides a significant inverse correlation between msBMR and MLS as a whole for mammals, it remains problematic for two reasons. First, several interspecies studies within respective orders (typically within rodents) have shown no inverse relationships between msBMR and MLS. Second, LEE values widely vary in mammals and birds. Here, to solve these two problems, we introduced a new quantity designated as mitochondrial (mt) lifetime energy output, mtLEO = MLS × mtMR, in place of LEE, by using the mt metabolic rate (mtMR) per mitochondrion. Thereby, we found that mtLEO values were distributed more narrowly than LEE ones, and strongly correlated with the four amino-acid variables (AAVs) of Ser, Thr and Cys contents and hydrophobicity of mtDNA-encoded membrane proteins (these variables were related to the stability of these proteins). Consequently, only these two mt items, mtMR and the AAVs, solved the above-mentioned problems in the rate-of-living theory, and thus extensively improved the correlation with MLS compared with that given by LEE.

## 1. Introduction

Longevity is one of the most fundamental measures of the activity of life. Ageing theories have proposed a number of environmental, lifestyle and genetic factors as the determinants of longevity, including calorie restriction [1], telomere length [2], insulin signalling [3], mitochondrial (mt) DNA mutations [4,5] and fatty-acid composition of membranes [6]. These theories may interact with each other in a complex way [7], and remain far from perfect because there are many unresolved controversies as well as contradictory observations [8–11].

Among ageing theories, the mt free-radical theory is one of the most well-known explanations of ageing [12] and is linked to the oxidative-damage theory [6,13,14]. The free-radical theory is derived from the longstanding rate-of-living theory that the product (lifetime energy expenditure, LEE) of maximum lifespan (MLS) and mass-specific basal metabolic rate (msBMR), LEE = MLS × msBMR, is approximately constant across many mammalian species [6,15]. The rate-of-living theory provides a significant inverse correlation between msBMR and MLS as a whole for mammals. However, it remains problematic for two reasons. First, several interspecies studies within respective orders have not shown such inverse relationships between MLS and msBMR [6,16]. As a typical example, the naked mole-rat has extreme longevity compared with other rodents of similar size, despite a high level of free radicals and significant levels of oxidative damage in proteins, lipids and DNAs [16–19]. Second, variations in LEE in mammals and birds exhibit diverse distribution patterns [20]. On the basis of these observations, researchers have argued that the rate-of-living theory cannot be correct, MLS is not a good marker of ageing, BMR is not a good measure of total energy metabolism [21] and reactive oxygen species are not causally linked with ageing [6,8].

Here, we clarified the cause of these problems in the rate-of-living theory and have proposed a solution to them. msBMR is probably proportional to the cell-specific metabolic rate (metabolic rate per unit cell, csMR; i.e. the standard cell metabolism), which is written as the product of the mt number per unit cell (mt density, *N*_mt_) and the mt metabolic rate (metabolic rate per mitochondrion, mtMR; i.e. csMR = *N*_mt_ × mtMR). Therefore, csMR and msMR depend on both *N*_mt_ and mtMR. Here, by using only mtMR, which represents the mt energy power, we introduced a new quantity referred to as the mt lifetime energy output (i.e. mtLEO = MLS × mtMR) in place of LEE and investigated the behaviour of this quantity by using a large number of animal data stored in the AnAge database [22]. As a result, the variation range of mtLEO in mammals and birds could be made smaller than that of LEE by adjusting a free parameter included in mtMR. This result provided a big clue to solve the main problem of the rate-of-living theory. More interestingly, we then found that mtLEO having the minimal variation range in mammals provided the maximal correlation with the four amino-acid variables (AAVs) of Ser, Thr and Cys contents (SC, TC and CC) and hydrophobicity (HYD) of mtDNA-encoded membrane proteins (MMPs). These four variables were previously shown to be related to the adaptive evolution associated with the stability of MMPs [23], although random AA substitutions are likely to cause the instability of them [24]. We observed that the correlation could be further improved by taking into account the phylogenetic effect of the same mtDNA sequences, without using other DNA sequences [21,25]. These two observations allowed us to identify mtMR with an energetic function of the same mt level as the AA sequences.

Consequently, only the two terms of mtMR and the four AAVs provided a surprisingly strong correlation (*R* = 0.96) with MLS, compared with the result (*R* = −0.66) of the rate-of-living theory, because they predicted different contributions to MLS in the respective animal groups. Especially, they formed sharply contrasting patterns of contribution in the rodent and cetacean lineages of quite small and large sizes, respectively.

Consequently, only two terms of mtMR and the four AAVs provided a surprisingly strong correlation (*R* = 0.96) with MLS, compared with the result (*R* = −0.66) of the rate-of-living theory, because they predicted different contributions to MLS in the respective animal groups. Especially, they formed sharply contrasting patterns of contribution in the rodent and cetacean lineages of quite small and large sizes.

## 2. Results

### 2.1. Expression of mtMR to introduce lifetime energy output

When msBMR used in the rate-of-living theory is proportional to the cell-specific metabolic rate (csMR = *N*_mt_ × mtMR), it depends on both *N*_mt_ and mtMR. We here report that only mtMR (which is the terminal unit of the cellular energy production and represents the mt energy power) can be strongly related to MLS (§4.1).

To define mtMR explicitly, we first used the allometric scaling law, BMR ≈ *a* × *M^b^*, with constants *a* and *b*, because this law provides a strong correlation (as *R* = 0.95) between the body mass (*M*) and BMR. We next changed the *b*-value so as to exactly reproduce the observed BMR data of each species. This could be done by putting *B* = ln(BMR/*a*)/ln(*M*). Then, we had msBMR = *a* × *M*^(*B*-1)^. We expressed mtMR for the mitochondrial metabolic rate as2.1
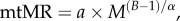
which changes the allometric scaling exponent of msBMR by using the free parameter *α* (*α* ≥ 1). mtMR can take a variety of *M*-dependences from *α* = 1 to *α* = ∞. It was therefore critical to determine the parameter *α* so that mtMR might be identified with the mt metabolic rate. This was done by introducing the new quantity of the mt lifetime energy output, mtLEO = MLS × mtMR, which was set so as to be equal to LEE at *α* = 1.

**Figure 1.**
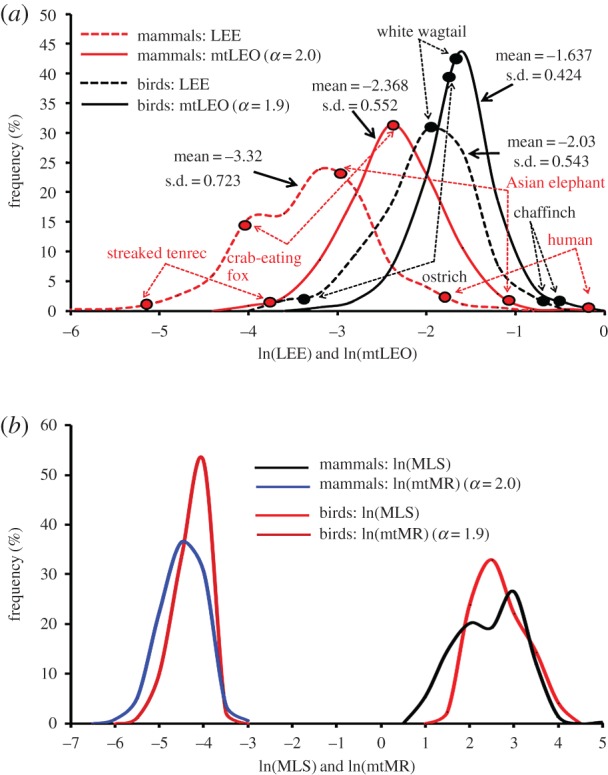
Frequency distributions of LEE and mtLEO in mammals and birds. LEE(w · yr/g) and mtLEO(w · yr/g) denote lifespan energy expenditure and mt lifetime energy output (equation (2.3)), respectively. The standard deviations (s.d.) of mtLEO were minimized at *α* ≈ 2.0 in both mammals and birds. The variations in mtLEO converged on narrower ranges than those in LEE (*a*). The mean mtLEO-value of birds was larger than that of mammals. Indeed, the MLS- and mtMR-distribution patterns of birds shifted more to the upper sides than those of mammals (*b*). The increase in MLS with *M* balanced the decrease in mtMR with *M*, but msBMR had a stronger *M*-dependence than mtMR. We plotted the positions of mtLEO and LEE on their distribution curves in (*a*). The positions largely shifted in the species with large body masses such as the Asian elephant in mammals and the ostrich in birds. Humans provided the largest mtLEO value.

We first followed the *α*-dependence of the standard deviation (s.d.) around the mean value of ln(mtLEO). We found that this s.d. could be minimized at *α* ≈ 2.0 in the case of both birds (167 samples) and mammals (347 samples). This result suggests that the rate-of-living theory is problematic. If this theory is justified, the minimization procedure of the mtLEO-variation has to select the condition *α* = 1 at which mtLEO is equal to LEE. Indeed, ln(mtLEO) showed narrow-range distributions (the solid curves in figure 1), in contrast with the wide-range distributions (the dotted curves in figure 1*a*) given by ln(mtLEO) at *α* = 1.0 (i.e. ln(LEE)): the s.d. was decreased by 22% in birds and 24% in mammals. The mean mtLEO value of birds was larger than that of mammals. Indeed, the MLS- and mtMR-distribution patterns of birds shifted more to the upper sides than those of mammals, respectively (figure 1*b*). The increase in MLS with *M* balanced the decrease in mtMR with *M*, whereas msBMR had a stronger *M*-dependence than mtMR.

We plotted the positions of mtLEO and LEE in several species on the distribution curves of them in figure 1*a*. The positions largely shifted in the species with large body masses such as the Asian elephant in mammals and the ostrich in birds. It is interesting to see that humans had the largest mtLEO value. The allometric scaling exponent (−0.125) of mtMR in mammals became similar to that (−0.12) of the mass-specific maximal metabolic rate [26].

### 2.2. Strong correlation between mtLEO and the 4 AA variables of MMPs

The second step to minimize the variation range of mtLEO was to relate mtLEO to the four AAVs (SC, TC, CC and HYD) of MMPs (the estimation method of these variable values is documented in §4.3), because our previous studies reported significant relationships between MLS and these variables in vertebrates and metazoans (no other AAVs significantly contributed to the correlation with MLS) [23,27]. We applied the Arrhenius-type equation to test the stability of protein material by tracing the temperature or humidity dependence of chemical reactions in this material [28,29]. We here extended this equation so as to describe a general multi-variable log-linear relationship that included different material properties (§4.2). We introduced the following equation for mtLEO = MLS × mtMR:2.2
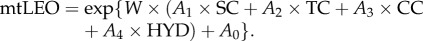
Here, linear regression analysis was possible in the logarithmic scale,2.3
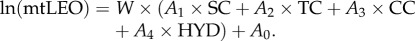


The four AAVs (SC, TC, CC and HYD) stand for different protein properties in the respective mammals. *W* denotes the branching weight in the phylogenetic analysis, given by inferring the tree structure from the complete AA sequences of all 13 proteins of MMPs (these sequences are available in electronic supplementary material ‘AMINO-ACID-SEQ’) [30].

**Figure 2.**
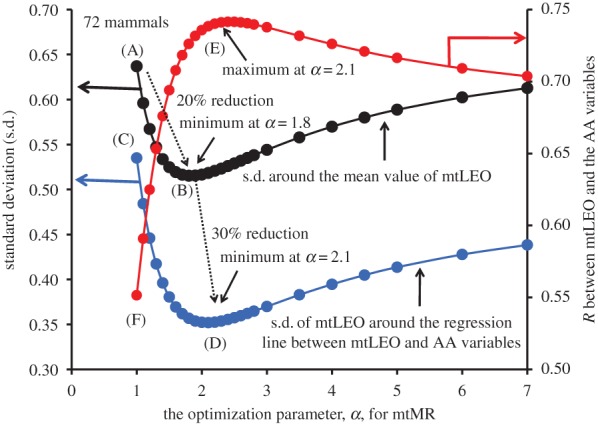
The *α-*dependence of the variation range of mtLEO. The black circles show the standard deviation (s.d., with the minimum at point ‘B’) around the mean value of mtLEO across species, whereas the blue circles show the s.d. (with the minimum at point ‘D’) around the regression line between mtLEO and the four AAVs. LEE corresponds to mtLEO at the initial point ‘A’. The correlation between mtLEO and the four AAVs has its maximum value at point ‘E’ (red circles).

We collected 72 mammals with species-to-species coincidence in both the AnAge database [22] and the NCBI genome database [31] (the coincidence extremely decreased the number of samples, especially in birds). We followed the *α*-dependence of the s.d. around the mean value of ln(mtLEO) by using these 72 samples (the black circles in figure 2). The s.d. was minimized at *α* = 1.8 (point ‘B’ in figure 2) and decreased by 20% compared with the original value (equal to the s.d. in ln(LEE)) at *α* = 1.0, i.e. point ‘A’). We finally followed the *α*-dependence of the s.d. in ln(mtLEO) around the regression line between ln(mtLEO) and the four AAVs (blue circles in figure 2). This s.d. provided one-step lower level values than the s.d. (black circles) around the mean value of ln(mtLEO) and was minimized at *α* = 2.1 (point ‘D’). The minimal value at point ‘D’ was further lowered to below the primary minimum at *α* = 1.8 (point ‘B’) by 30%. As a result, the correlation between mtLEO and the four AAVs was maximized at the same *α* = 2.1 (point ‘E’; red circles in figure 2, in which point ‘F’ gives a lower correlation in the case of mtLEO = LEE at *α* = 2.1). The appearance of this maximum correlation strongly suggests that the obtained mtMR was the energetic function belonging to the same mitochondrial level as the four AAVs.

**Figure 3.**
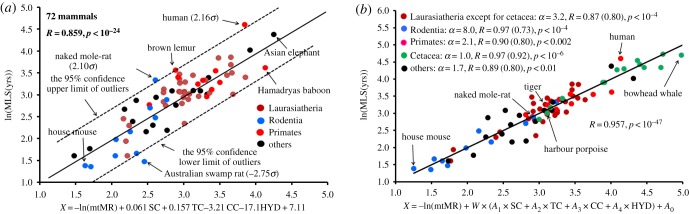
MLS expressed in terms of mtMR and the AAVs in 72 mammals. (*a*) The standard regression analysis with *W* = 1.0 and the optimized mtMR (*α* = 2.1). *σ* denotes the standard deviation around the regression line, and is shown for species that deviated from the 95% confidence limit of the regression line. The variable values used in this analysis are listed in electronic supplementary material, table S1. (*b*) Stratified phylogenetic regression analysis. The values in parentheses stand for the *R*-values without the phylogenetic effect (*W* = 1.0 in equation (2.3)). The *α*-values were given by optimizing mtMR in the respective animal groups. The coefficients (*Ai*, *i* = 0–4) are as follows: (i) Laurasiatheria, *A*_1_ = 1.0955, *A*_2_ = −0.2298, *A*_3_ = −9.8398, *A*_4_ = −1.4322, *A*_0_ = −1.7201; (ii) Rodentia, *A*_1_ = 1.0289, *A*_2_ = 2.3849, *A*_3_ = −4.1904, *A*_4_ = −42.0586, *A*_0_ = −3.2168; (iii) Primates, *A*_1_ = −0.4466, *A*_2_ = 0.6752, *A*_3_ = −1.5907, *A*_4_ = −1.6315, *A*_0_ = −2.5397; (iv) Cetacea, *A*_1_ = −0.0429, *A*_2_ = −0.1714, *A*_3_ = 0.2435, *A*_4_ = 4.0870, *A*_0_ = −6.9390; (v) others, *A*_1_ = 0.9514, *A*_2_ = 0.2522, *A*_3_ = −1.2723, *A*_4_ = −16.1261, *A*_0_ = −2.0287. The other variable values used in this analysis are listed in electronic supplementary material, table S2.

When the coefficients *A_i_* (*i* = 0–4) of the four AAVs were determined by the multi-variate regression analysis of equation (2.3) (using mtMR at *α* = 2.1), MLS was finally given by the following equation:2.4
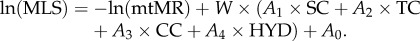
Here, MLS was well expressed in terms of mtMR and the four AAVs, with *R* = 0.86 and *p* < 10^−24^ (figure 3*a*). The positive correlation (*R* = 0.67) between MLS and the four AAVs was compatible with the inverse correlation (*R* = −0.66) between MLS and mtMR (table 1). As shown in the *x*-axis of figure 3*a*, SC and TC increased with increasing MLS, whereas CC and HYD decreased with increasing MLS. This behaviour is an important signal for the stability of MMPs (§§3.4 and 3.5). We here selected six large subunits (ND4, ND5, ND2, CO1, CO3 and CYTB) from complexes I, IV and III, as providing significant correlations with ln(mtLEO). These subunits covered 68% of the complete AA sequences (4074 sites) and constituted the bulk of the proton-pump machinery of the respiratory chain.

**Table 1.** Correlation coefficients (*R*) for several sets of the variables. SC, TC, CC and HYD denote the AAVs of Ser, Thr, Cys contents and hydrophobicity, respectively. *p*- and *F*-values for the *F*-statistics and AIC are given. There was no significant correlation between mtMR and the four AAVs. The values in bold indicate the largest correlation coefficients.

	ln(MLS)	ln(mtLEO)	ln(LEE)
SC, TC, -HYD	0.46 (*p* < 10^−3^)*F* = 6, AIC = 142	0.50 (*p* < 5.0 × 10^−4^) *F* = 8, AIC = 99	0.42 (*p* < 5.0 × 10^−3^) *F* = 5, AIC = 135
SC, TC, -CC	0.62 (*p* < 10^−6^) *F* = 14, AIC = 124	0.71 (*p* < 10^−9^) *F* = 23, AIC = 69	0.55 (*p* < 10^−5^) *F* = 10, AIC = 124
HYD, -CC	0.64 (*p* < 10^−7^) *F* = 24, AIC = 120	0.73 (*p* < 10^−11^) *F* = 38, AIC = 64	0.48 (*p* < 10^−4^) *F* = 11, AIC = 128
-CC	0.54 (*p* < 10^−6^) *F* = 29, AIC = 130	0.62 (*p* < 10^−8^) *F* = 43, AIC = 81	0.41 (*p* < 5 × 10^−4^) *F* = 8, AIC = 132
SC, TC, -HYD, -CC	**0.67 (*p* < 10^−9^)** *F* = 18, AIC = 119	**0.74 (*p* < 10**^−**13**^**)** *F* = 27, AIC = 65	**0.55 (*p* < 10**^−**5**^**)** *F* = 10, AIC = 125
-ln(mtMR)	**0.66 (*p* < 10**^−**9**^**)** *F* = 53, AIC = 115	―	―
-ln(mtMR), SC, TC, -HYD, -CC	**0.86 (*p* < 10**^−**24**^**)** *F* = 64, AIC = 67	―	―

There appeared three species (Australian swamp rat, human and naked mole-rat) as outliers outside the 95% confidence limit of the regression line (figure 3*a*). Table 1 gives the correlation coefficients of several sets of variables and provides the *p*- and *F*-values for the *F*-statistics and the Akaike information criterion (AIC). At this stage, we simply performed the standard regression analysis, setting *W* = 1.0 (figures 2 and 3*a*). The values of variables used in this analysis are listed in electronic supplementary material, table S1.

### 2.3. Sharply contrasting behaviours in the rodent versus cetacean lineages

**Figure 4.**
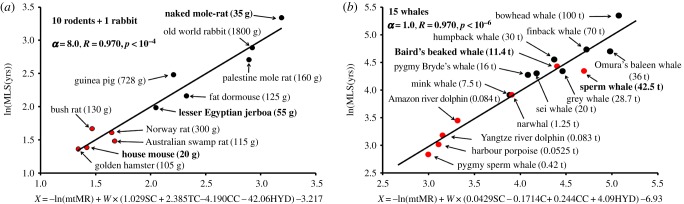
Phylogenetic analysis of the rodent and cetacean lineages. (*a*) Rodent analysis. The optimization procedure given by adjusting *α* in equation (2.3) provided mtMR ≈ constant at *α* = 8.0. The variable values used in this analysis are listed in electronic supplementary material, table S2. (*b*) Cetacean analysis. The optimization procedure given by adjusting *α* in equation (2.3) provided mtMR = msBMR with *α* = 1.0. The red and black circles show the toothed and baleen whales, respectively. The variable values used in this analysis are listed in electronic supplementary material, table S2.

To clarify the reason for why the naked mole-rat has an extremely long lifespan compared with other rodents of similar size, we performed a stratified regression analysis of the rodent lineage. Minimizing the s.d. in ln(mtLEO) around the regression line between ln(mtLEO) and the four AAVs in equation (2.3) gave a large value of *α* = 8.0. Then we obtained a strong correlation between MLS and mtMR plus the four AAVs, with *R* = 0.97 and *p* < 10^−4^ (figure 4*a*). Here, mtMR with a small value of the allometric scaling exponent at this large *α* value contributed little to this correlation. In fact, the scatter plots of the respective species were located independent of *M*. The naked mole-rat (35 g) was positioned at the highest level due to a predominant contribution of the AAVs to MLS, whereas the house mouse (20 g) was positioned at the second lowest level. Here, we included the Old World rabbit (1800 g) for reference; however, the MLS of this species was lower than that of the naked mole-rat, despite a much larger body mass. The contribution of CC to MLS was small (indeed, excluding mtMR and CC still gave a strong correlation of *R* = 0.93).

We took into account the branching weight *W* in equation (2.3) on the basis of the phylogenetic procedure [30]. This weight effect was important because setting *W* = 1.0 yielded a small value of *R* = 0.72. The complete AA sequences of all 13 proteins of MMPs were necessary for a robust tree building. In this sense, it was meaningful to use the same DNA sequences for both the tree building and the four AAVs, different from previous studies using other DNA sequences [21,25]. Indeed, the tree structure (electronic supplementary material, figure S1) was clearly separated into two groups of short MLS values (rat, mouse and hamster) and long MLS values (the others; figure 4*a*).

The phylogenetic analysis of the cetacean lineage exhibited different behaviours in contrast with the rodent lineage. Minimizing the s.d. in ln(mtLEO) around the regression line between ln(mtLEO) and the four AAVs gave the lowest value of *α* = 1.0. As a result, we obtained a strong correlation between MLS and mtMR plus the four AAVs, with *R* = 0.94 and *p* < 10^−6^ (figure 4*b*). Here, mtMR rapidly decreased with increasing *M* and showed a strong inverse correlation (*R* = 0.92) with MLS. The correlation between the four AAVs and MLS was rather small (*R* = 0.67), because of the predominant contribution of mtMR to MLS. SC and TC steadily decreased with increasing MLS, whereas CC and HYD increased with increasing MLS, in sharp contrast with the behaviours of terrestrial mammals (figure 3*a*). This behaviour was a striking feature of the cetacean lineage, in which animals returned from land to water and evolved from toothed whales to baleen whales through the buoyancy effect. As shown in figure 4*b*, the sperm whale (*Physeter macrocephalus*) and Baird's beaked whale (*Berardius bairdii*), which are toothed whales that dive into the deep sea and have huge bodies, were included in the baleen whale group.

### 2.4. Stratified regression analysis as a whole for mammals

We classified mammals into the following five groups: (i) Rodentia (Glires including rabbits; 11 species), (ii) Cetacea (15 species), (iii) Primates (10 species), (iv) Laurasiatheria (excluding cetaceans with 33 species), and (v) others (17 species including Afrotheria, Xenarthra and Marsupial). Figure 3*b* shows that the stratified phylogenetic analysis of these respective mammal groups provided a better result (*R* = 0.96 and *p* < 10^−47^) compared with the one-step regression analysis for mammals as a whole (figure 3*a* with *R* = 0.86). The variable values used in this analysis are listed in electronic supplementary material, table S2.

The correlations in the respective animal groups were always improved by the phylogenetic analysis, reflecting the adaptive evolution for the four AAVs compared with the *R* values without taking into account the evolutionary pathway (figure 3*b*). Here, the stratified analysis was useful to suppress the influence of the long-branch attraction (convergent evolution) in the tree building procedure of the AA sequences [32]. The phylogenetic effect was especially large in the rodent lineage owing to a large contribution of the four AAVs to MLS and small in the cetacean lineage owing to a small contribution of them to MLS.

### 2.5. Evidences to identify the obtained mtMR with mt metabolism

The first evidence was that mtLEO with the minimal variation range provided the maximal correlation with the four AAVs (SC, TC, CC and HYD), which can be related to the stability of MMPs (§§3.4 and 3.5). Then, the obtained mtMR could be regarded as the energetic function belonging to the same mitochondrial level as the four AAVs. The second evidence was given by the stratified regression analysis. The phylogenetic weight factor was useful for definitively determining the different *α*-values in the respective animal groups and improving the correlation with MLS (figure 3*b*).

For example, minimization of the mtLEO variation in the rodent lineage provided a very high value of *α* = 8.0, at which mtMR had a very low dependence on *M*. Consequently, only the four AAVs predominantly contributed to MLS and solved the long-standing MLS problem between the house mouse and the naked mole-rat in the rate-of-living theory. On the other hand, minimization of the mtLEO variation in the cetacean lineage including baleen whales of great size provided the lowest value of *α* = 1.0 at which mtMR rapidly decreased with increasing *M*. Consequently, the multi-cellular effect of metabolism owing to body mass predominantly contributed to MLS. In this way, the extremely different *α*-values in the two lineages were quite reasonable to explain the observed MLS data.

## 3. Discussion

### 3.1. Validity of the minimization approach of the mtLEO variation

This minimization approach given by using equation (2.3) was very useful for (i) clarifying the reason for why the long-standing rate-of-living theory is problematic, (ii) solving this problem by excluding the mt density (*N*_mt_) term included in msBMR, and (iii) clarifying the reason for why the remaining term mtMR in msBMR could be identified with the mt metabolic rate. We here emphasize that this approach is quite robust and not special in any way. Indeed, by directly solving equation (2.4), in which the first term was rewritten as *A*_5_ × ln(mtMR), we could obtain *A*_5_ = −1.0. Also, by rewriting this term as *A*_5_ × ln(msBMR), we obtained *A*_5_ = −1.0/*α* and the same correlation *R* coefficients and *A_i_* (*i* = 1–4) except for a change in *A*_0_.

### 3.2. Schematic picture of mammalian longevity in terms of mtMR and the AA variables

**Figure 5.**
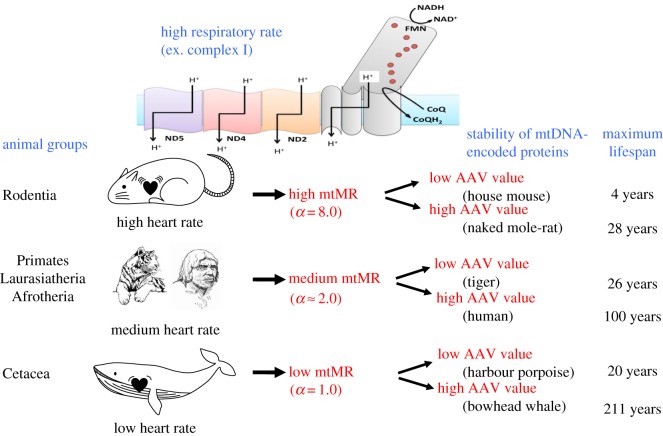
Present view of mammalian longevity. Maximum life span (MLS) is expressed as the balance between the mt metabolic rate (mtMR) and the AAV in the respective animal groups and species, because equation (2.2) can be rewritten as MLS = exp(AAV)/mtMR with AAV = *W* × (*A*_1_ × SC + *A*_2_ × TC + *A*_3_ × CC + *A*_4_ × HYD) + *A*_0_. Here, SC, TC and CC and HYD stand for Ser, Thr and Cys contents and hydrophobicity of mtDNA-encoded membrane proteins, respectively. Here, AAV and mtMR are related to the stability of MMPs and the multi-cellular effect depending on the body mass (*M*), respectively. The proton translocation in the mt inner membrane requires dynamic and large conformational changes involving several mtDNA-encoded protein subunits.

The present view of mammalian longevity is shown in figure 5. As easily understood in equation (2.2), the MLS depends on the combination (balance) between mtMR and the AAV: MLS = exp(AAV)/mtMR with AAV = *W* × (*A*_1_ × SC + *A*_2_ × TC + *A*_3_ × CC + *A*_4_ × HYD) + *A*_0_. Here, AAV is associated with the stability of MMPs, whereas mtMR represents the multi-cellular effect based on the body mass (*M*; as discussed in the following sections). The proton translocation in the mt inner membrane requires dynamic and large conformational changes in MMPs. Therefore, relatively larger AAV and smaller mtMR makes the stability of MMPs stronger.

In the rodent lineage with very high mtMR, mice and rats of small size have very short MLS (4 years) owing to their lower AAV, whereas the naked mole-rat, which is of similar size, has a long MLS (28 years) owing to its higher AAV. On the other hand, the bowhead whale, of great size, has a very long MLS (200 years) owing to its very low mtMR. The MLS of other animals varied depending on a variety of combinations between mtMR and AAV. Humans seem to have realized an optimal balance between them, so as to attain both high mitochondrial activity and long longevity. The exp(AAV) and mtMR values of several species are listed in table 2. Although these values greatly differed across species from the house mouse to the bowhead whale, their ratios remained within a small range to reproduce MLS.

**Table 2.** Mitochondrial determinants of MLS. Equation (2.2) was rewritten as MLS = exp(AAV)/mtMR with AAV = *W* **×** (*A*_1_ **×** SC + *A*_2_ **×** TC + *A*_3_ **×** CC + *A*_4_ **×** HYD) + *A*_0_. Here, the values of exp(AAV) and mtMR of seven species as typical examples of mammals are listed (they were determined by the regression analysis of equation (2.3)). Although these values greatly differ across species, the ratios of them remained within a small range.

species	MLS (years)	*M*	exp(AAV)	mtMR (arb units)	exp(AAV)/mtMR
house mouse	4	20 g	0.0492	0.0143	3.4
naked mole-rat	28	35 g	0.3167	0.0122	26.0
tiger	26	12 kg	0.1412	0.0065	21.7
human	100	70 kg	0.3068	0.0044	70.0
harbour porpoise	20	53 kg	0.00526	0.000233	22.6
bowhead whale	211	100 t	0.00204	0.000013	154.7

### 3.3. Biological behaviours specific to the respective animal groups

Vertebrates that had undergone the water-to-land transition developed their skeletons, muscles and aerobic capacities to move on dry land. This transition necessitated the consumption of a large amount of cellular energy.

First of all, it is both interesting and instructive to understand the evolutionary behaviour of cetaceans that returned from land to water, regained both buoyancy and zero-gravity environments, and evolved from toothed whales to baleen whales that are huge in size. The stratified regression analysis minimizing the mtLEO variation predicted *α* = 1.0 for mtMR. As a result, mtMR exhibited ‘a rapid decrease’ accompanied by an increase in *M* with a strong correlation with MLS (*R* = −0.92). As explained in the next section, the decrease in mtMR reduced the dynamic conformational changes and mobility of MMPs, and increased their stability by the trade-off between mobility and stability in the membrane protein folding problem [33,34]. This stabilization could be enhanced by increasing the HYD of the proteins owing to their being surrounded by lipid bilayers. In this way, the behaviour of mtMR associated with the multi-cellular effect may be considered to play a basic role in prolonging the lifespan of not only mammals but also metazoans in general.

By contrast, terrestrial mammals exhibited an average value *α* ≈ 2.1 for mtMR, which decreased more slowly than that in cetaceans with increasing *M*. These mammals had to enlarge their aerobic capacity with increasing *M* in order to move against gravity. For this purpose, it was necessary to enhance the potential for mitochondrial energy production; and so higher dynamic conformational changes in MMPs were required. The mobility of MMPs was increased by decreasing HYD, their stability through helix–helix interactions was enhanced by increasing SC and TC, and CC was decreased so as to reduce the oxidative damage induced by the larger aerobic capacity. These behaviours sharply contrasted with those of the cetacean lineage. As a result, mtMR and the four AAVs provided mutually compatible contributions to MLS.

The rodent lineage showed specific features with *α* = 8.0 for mtMR. Then, mtMR became almost independent of *M* (electronic supplementary material, figure S2). Small animals, such as mice and rats, may have a lower limit to the size of their main organs (in which the cells include a large number of mitochondria), such as the heart, liver and kidney. Thermogenesis is an important factor that allows rodents to maintain their body temperature, despite their small sizes [35]. This would require leakage of a large amount of protons through uncoupling proteins [36]. For this purpose, both decreased HYD and increased SC and TC are useful for highly activated dynamic conformational changes in respiratory chain proteins to enhance the proton pumping power (as discussed in the next section). Consequently, MLS was well reproduced by only these three AAVs (*R* = 0.93); and no significant correlation between CC and MLS was observed. The present result is interesting, because recent studies have focused on providing an explanation for the extreme longevity of the naked mole-rat [6,17,18]. We understand that evolutionary behaviours of rodents are specific and largely different from those of other terrestrial mammals.

### 3.4. Amino acid variables underlying stability of MMPs

Recent structural studies on MMPs have suggested that proton translocation in complex I requires dynamic and large conformational changes involving several mtDNA-encoded protein subunits [37–40]. Similarly, the two large subunits of complex IV (CO1 and CO3) transfer protons across the membrane via conformational changes induced by electron transport [41–43]. In addition, mitochondrial morphologies can change dramatically by shifting the balance between fusion and fission, which plays an essential role for mitochondrial activity in an organism [44]. Fusion helps mitigate stress by mixing the contents of partially damaged mitochondria as a form of complementation. Fission is needed to create new mitochondria, but it also contributes to quality control by enabling the removal of damaged mitochondria and can facilitate apoptosis during high levels of cellular stress. In this way, MMPs may undergo large movement during their function.

The stability of MMPs can be explained well based on the membrane-protein folding problem [33,34]. Notably, membrane proteins have the unusual ability to strengthen their dynamic stability through inter-helical interactions between motifs involving moderately polar residues, such as Ser and Thr [34,45,46]. Therefore, increases in SC or TC are likely to correspond to increased hydrogen bonding between helices, both within and between subunits. A decrease in HYD may facilitate these conformational changes by increasing mobility in MMPs, which are embedded in the hydrophobic environment of the lipid bilayer (§4.3). Importantly, the dynamic stability provided by inter-helical interactions predominates over the instability induced by increasing mobility. In fact, increases in MLS were associated with increases in SC and TC and decreases in HYD, as was shown in the *x*-axis of figure 3.

### 3.5. Oxidative damage effect induced by the CC variable

The mitochondrial free-radical theory of ageing has been recently refuted, because the age-related increases in oxidative damage and ROS production are relatively small [7]. The main problem with this theory is typically demonstrated in the naked mole-rat, which has extreme longevity compared with other rodents of similar size, despite a high level of free radicals and significant levels of oxidative damage in proteins, lipids and DNA [16–19]. However, a global viewpoint of metazoans is that CC sensitivity to oxidative damage is significantly correlated with MLS [25,47]. We also observed an inverse correlation between CC and MLS for terrestrial mammals as a whole (table 1). We here note that CC is one of the determinants of MLS and that interspecies within respective orders (typically within rodents) may not necessarily show a significant inverse correlation between it and MLS, suggesting that rodents therefore followed the life strategy of rapidly increasing their population in a short life span and that the time interval (MLS) to accumulate oxidative damage is important. We note that the cetacean lineage showed no oxidative damage effect by CC.

## 4. Material and methods

### 4.1. Reason for employing the mitochondrial metabolic rate

The mass-specific metabolic rate (msMR) is probably proportional to the csMR, which can be written as csMR = *N*_mt_ × mtMR in terms of the mt density (*N*_mt_) and mtMR per mitochondrion. Here, *N*_mt_ is likely to decrease with increasing *M* owing to the multi-cellular effect [48,49]. However, the behaviour of mtMR remains unclear. The fractal-like network model for the allometric scaling law assumes that mtMR is invariant as the terminal unit of the network [49]. However, when both *N*_mt_ and mtMR change across species, csMR (also msMR) becomes confusing or ambiguous. Recent structural studies on MMPs have shown that the respiratory chain has a high degree of sequence conservation in the membrane integral central subunits [50]; therefore, its mechanism is likely to be similar across species [37]. However, we recently demonstrated that the four AAVs of SC, TC, CC and HYD of MMPs are highly variable across species and can be related to MLS [20,27]. A significant correlation between CC and MLS in metazoans has also been reported [25,47]. These results strongly suggest that mitochondrial functions may be influenced by the adaptive evolution of respective species and that both *N*_mt_ and mtMR may change across species.

### 4.2. Extension of the Arrhenius-type equation for mtLEO

The Arrhenius life stress model has been widely used to investigate the quality of a fixed material as a function of temperature and humidity and assumes that the product of lifetime (LT) and reaction rate (RR) is a constant, just as in the rate-of-living theory. Therefore, RR can be written as RR = exp(−*T*_0_/*T* + *H*_0_/*H* + *C*) with temperature *T*, humidity *H* and constants *T*_0_ and *C* [28,29]. The stability of the material is estimated as being proportional to 1/RR. In this study, we extended the Arrhenius-type equation to explicitly take into account differences in various materials: thereby, we obtained the expression shown in equation (2.2). On the basis of the concept of the Arrhenius equation, equation (2.2) assumes that the turnover time of mitochondria is proportional to MLS of an organism via that of cells and organs, because this equation describes the chemical reactions within MMPs.

### 4.3. Estimation of the four amino acid variables (SC, TC, CC and HYD)

To select hydrophobic domains (the helix region) in MMPs, we performed primary sequence analyses using a standard model for the hydrophobic score (*S*) of AAs by using a standard model for the hydrophobic score (*S*) of AAs [51,52]. The moving average *S*_av_ of local hydrophobic score *S* around each AA was calculated for HYD (the mean value of *S*_av_ about all sequences). We calculated the total numbers of the respective AAs within the hydrophobic domains and the relative contents (%) of them to obtain SC, TC and CC. The strongest correlation between mtLEO and AAVs was obtained by selecting AA sites with *S*_av_ > 1.5.

## Supplementary Material

Table S1. Basic data for the present analysis found in Figs. 1-3

## Supplementary Material

Table S2. Basic data for the stratified analysis found in Figs. 4-5

## Supplementary Material

Figure S1. Rodent phylogeny.

## Supplementary Material

Figure S2. Phylogenetic analysis of the rodent lineage.

## Supplementary Material

AMINO-ACID-SEQ

## Data accessibility

The datasets supporting this article have been uploaded as part of the electronic supplementary material (as the five files of electronic supplementary material, tables S1 and S2, figures S1 and S2 and AMINO-ACID-SEQ).
