# Supplementary material for: Mitochondrial determinants of mammalian longevity
Source: Open Biol. 2017 Oct 25;7(10):170083. doi: 10.1098/rsob.170083 (PMC5666079; doi:10.1098/rsob.170083)
Supplement: Table S1. Basic data for the present analysis found in Figs. 1-3 [file rsob170083supp1.docx]

**Table S1**. **Basic data for the present analysis shown in Figs. 1-3**

| NCBI | Order | Species | ln(*M*) | ln(*MLS****)*** | ln(*mtMR*) | SC(%) | *TC*(%) | *CC*(%) | *HYD* |
| --- | --- | --- | --- | --- | --- | --- | --- | --- | --- |
| 06853 | Ar | *Bos taurus* | 13.528 | 3.401 | -5.934 | 7.140 | 8.117 | 0.5525 | 0.5362 |
| 09849 | Ar | *Camelus dromedarius* | 12.981 | 3.346 | -5.822 | 7.229 | 7.960 | 0.6885 | 0.5351 |
| 07704 | Ar | *Cervus elaphus* | 12.206 | 3.450 | -5.783 | 7.134 | 8.209 | 0.5558 | 0.5425 |
| 07703 | Ar | *Rangifer tarandus* | 11.525 | 3.077 | -5.429 | 7.167 | 8.100 | 0.6361 | 0.5445 |
| 00845 | Ar | *Sus scrofa* | 12.101 | 3.045 | -5.457 | 7.220 | 7.955 | 0.6485 | 0.5362 |
| 15889 | Ar | *Ovis canadensis* | 11.160 | 3.025 | -5.275 | 6.974 | 8.707 | 0.5917 | 0.5402 |
| 15247 | Ar | *Odocoileus virginianus* | 11.374 | 3.073 | -5.342 | 7.255 | 8.146 | 0.6364 | 0.5391 |
| 12103 | Ar | *Lama glama* | 9.913 | 3.450 | -5.272 | 7.300 | 8.942 | 0.6479 | 0.5302 |
| 12102 | Ar | *Pecari tajacu* | 11.849 | 3.364 | -5.479 | 7.430 | 7.771 | 0.6832 | 0.5360 |
| 01788 | Pe | *Equus asinus* | 12.087 | 3.850 | -5.543 | 7.699 | 8.169 | 0.5560 | 0.5370 |
| 05212 | Ca | *Acinonyx jubatus* | 10.887 | 3.020 | -5.440 | 7.242 | 8.556 | 0.5506 | 0.5218 |
| 09691 | Ca | *Ailurus fulgens* | 8.372 | 2.944 | -5.449 | 7.048 | 8.116 | 0.5980 | 0.5491 |
| 08093 | Ca | *Canis latrans* | 9.492 | 3.082 | -5.326 | 7.137 | 7.954 | 0.6019 | 0.5390 |
| 09692 | Ca | *Enhydra lutris* | 10.197 | 3.296 | -5.192 | 7.230 | 8.584 | 0.5497 | 0.5285 |
| 09685 | Ca | *Gulo gulo* | 9.701 | 2.970 | -5.192 | 7.085 | 8.273 | 0.5515 | 0.5317 |
| 06835 | Ca | *Herpestes javanicus* | 6.415 | 2.809 | -4.888 | 7.361 | 8.050 | 0.5596 | 0.5248 |
| 09970 | Ca | *Melursus ursinus* | 11.513 | 3.506 | -5.867 | 7.207 | 8.022 | 0.6006 | 0.5336 |
| 01325 | Ca | *Phoca vitulina* | 11.525 | 3.077 | -5.429 | 7.549 | 8.227 | 0.5937 | 0.5303 |
| 09126 | Ca | *Procyon lotor* | 8.700 | 3.045 | -5.245 | 6.911 | 8.423 | 0.6048 | 0.5371 |
| 08434 | Ca | *Vulpes vulpes* | 8.326 | 3.059 | -4.936 | 7.622 | 7.536 | 0.6063 | 0.5300 |
| 14456 | Ca | *Lynx rufus* | 9.060 | 3.475 | -5.031 | 6.964 | 8.110 | 0.5945 | 0.5279 |
| 01325 | Ca | *Phoca vitulina* | 11.653 | 3.863 | -5.723 | 7.549 | 8.227 | 0.5937 | 0.5303 |
| 10497 | Ca | *Spilogale putorius* | 6.397 | 2.351 | -5.020 | 7.471 | 0.158 | 0.6011 | 0.5242 |
| 11358 | Ca | *Lutra lutra* | 8.817 | 2.901 | -4.882 | 7.143 | 8.846 | 0.5527 | 0.5273 |
| 10642 | Ca | *Panthera tigris* | 11.693 | 3.270 | -5.455 | 7.240 | 8.220 | 0.7240 | 0.5304 |
| 09126 | Ca | *Procyon lotor* | 8.700 | 3.045 | -5.245 | 6.911 | 8.423 | 0.6048 | 0.5371 |
| 02009 | Ch | *Artibeus jamaicensis* | 3.742 | 2.303 | -4.489 | 7.194 | 8.591 | 0.5501 | 0.5504 |
| 02619 | Ch | *Pteropus scapulatus* | 5.704 | 1.609 | -4.773 | 7.528 | 7.956 | 0.5560 | 0.5328 |
| 05434 | Ch | *Pteropus pumilus* | 5.298 | 2.845 | -4.908 | 7.388 | 8.419 | 0.5584 | 0.5447 |
| 07393 | Ch | *Rousettus egyptiacus* | 4.828 | 3.131 | -4.699 | 7.340 | 8.443 | 0.5515 | 0.5336 |
| 02808 | Eu | *Echinosorex gymnura* | 7.131 | 1.609 | -5.122 | 7.546 | 7.588 | 0.7206 | 0.5491 |
| 02080 | Eu | *Erinaceus europaeus* | 6.620 | 2.460 | -4.948 | 8.156 | 6.839 | 0.6797 | 0.5449 |
| 05033 | Eu | *Hemiechinus auritus* | 5.988 | 1.946 | -5.150 | 6.955 | 7.732 | 0.7775 | 0.5405 |
| 00884 | Ro | *Cavia porcellus* | 6.590 | 2.485 | -4.997 | 7.948 | 8.078 | 0.6911 | 0.5306 |
| 05314 | Ro | *Jaculus jaculus* | 4.007 | 1.988 | -4.443 | 8.219 | 7.920 | 0.6421 | 0.5385 |
| 05089 | Ro | *Mus musculus* | 3.020 | 1.386 | -4.279 | 7.549 | 7.809 | 0.6508 | 0.5489 |
| 01892 | Ro | *Myoxus glis* | 4.828 | 2.163 | -4.713 | 8.235 | 8.017 | 0.6536 | 0.5416 |
| 005315 | Ro | *Nannospalax ehrenbergi* | 5.075 | 2.708 | -4.891 | 7.815 | 8.377 | 0.6908 | 0.5309 |
| 001665 | Ro | *Rattus norvegicus* | 5.704 | 1.609 | -4.773 | 7.669 | 8.057 | 0.7324 | 0.5409 |
| 015112 | Ro | *Heterocephalus glaber* | 3.555 | 3.343 | -4.891 | 8.480 | 7.878 | 0.6888 | 0.5247 |
| 014858 | Ro | *Rattus lutreolus* | 4.745 | 1.482 | -4.974 | 7.830 | 8.091 | 0.6960 | 0.5370 |
| 014867 | Ro | *Rattus fuscipes* | 4.868 | 1.668 | -4.895 | 7.879 | 7.836 | 0.6926 | 0.5369 |
| 013276 | Ro | *Mesocricetus auratus* | 4.654 | 1.361 | -4.612 | 7.756 | 7.799 | 0.7799 | 0.5404 |
| 001913 | La | *Oryctolagus cuniculus* | 7.496 | 2.890 | -4.835 | 7.438 | 8.134 | 0.5220 | 0.5422 |
| 06901 | Pr | *Colobus guereza* | 9.205 | 3.555 | -5.252 | 7.033 | 9.676 | 0.5968 | 0.5257 |
| 01807 | Pr | *Homo sapiens* | 11.156 | 4.605 | -5.428 | 7.621 | 9.554 | 0.5570 | 0.5211 |
| 01992 | Pr | *Papio hamadryas* | 9.798 | 3.624 | -5.433 | 7.168 | 10.544 | 0.5277 | 0.5177 |
| 02765 | Pr | *Nycticebus coucang* | 6.592 | 3.250 | -5.259 | 7.868 | 8.856 | 0.6449 | 0.5270 |
| 12766 | Pr | *Eulemur fulvus* | 8.055 | 3.570 | -5.367 | 7.297 | 8.358 | 0.7213 | 0.5305 |
| 12775 | Pr | *Saimiri sciureus* | 6.830 | 3.408 | -4.763 | 7.326 | 8.870 | 0.5296 | 0.5338 |
| 12774 | Pr | *Tarsius syrichta* | 4.781 | 2.773 | -4.897 | 7.434 | 8.454 | 0.6797 | 0.5333 |
| 12764 | Pr | *Perodicticus potto* | 7.111 | 3.288 | -5.288 | 7.783 | 9.130 | 0.6522 | 0.5117 |
| 12763 | Pr | *Loris tardigradus* | 5.473 | 2.960 | -4.986 | 8.402 | 8.751 | 0.5660 | 0.5194 |
| 12762 | Pr | *Otolemur crassicaudatus* | 6.998 | 3.122 | -5.097 | 7.837 | 8.773 | 0.5537 | 0.5225 |
| 05280 | Ce | *Phocoena phocoena* | 10.869 | 3.016 | -5.166 | 6.946 | 9.276 | 0.5083 | 0.5403 |
| 02631 | Af | *Echinops telfairi* | 5.193 | 2.944 | -4.829 | 7.860 | 7.692 | 0.5017 | 0.5517 |
| 06924 | Pi | *Cyclopes didactylus* | 5.561 | 3.332 | -5.082 | 7.125 | 9.9142 | 0.6009 | 0.5280 |
| 04032 | Pi | *Tamandua tetradactyl* | 8.412 | 2.197 | -5.457 | 7.085 | 7.891 | 0.7213 | 0.5378 |
| 04026 | Ma | *Macro. proboscideus* | 3.689 | 2.163 | -4.562 | 7.762 | 8.716 | 0.6505 | 0.5253 |
| 02078 | Tu | *Orycteropus afer* | 11.002 | 3.395 | -5.775 | 7.995 | 7.367 | 0.5860 | 0.5465 |
| 10302 | Si | *Trichechus manatus* | 12.682 | 4.025 | -6.235 | 7.679 | 8.065 | 0.6006 | 0.5425 |
| 05129 | Ci | *Elephas maximus* | 15.116 | 4.382 | -5.723 | 7.385 | 9.383 | 0.4778 | 0.5277 |
| 01821 | Ci | *Dasypus novemcinctus* | 8.613 | 3.105 | -5.588 | 7.287 | 8.658 | 0.6858 | 0.5386 |
| 07630 | Di | *Potorous maculatus* | 7.265 | 2.674 | -5.232 | 7.076 | 8.483 | 0.8525 | 0.5396 |
| 06519 | Di | *Pseudo. peregrinus* | 6.745 | 2.313 | -5.041 | 7.375 | 8.373 | 0.6941 | 0.5353 |
| 03039 | Di | *Trichosurus vulpecula* | 7.781 | 2.766 | -5.288 | 7.639 | 8.157 | 0.6905 | 0.5307 |
| 01794 | Di | *Macropus robustus* | 10.309 | 3.091 | -5.462 | 7.255 | 8.104 | 0.7213 | 0.5366 |
| 08134 | Di | *Petaurus breviceps* | 4.700 | 2.879 | -4.771 | 7.619 | 8.182 | 0.6926 | 0.5323 |
| 08133 | Di | *Phascolarctos cinereus* | 8.615 | 3.096 | -5.489 | 7.246 | 10.039 | 0.7857 | 0.5208 |
| 06523 | Da | *Phascogale tapoatafa* | 5.265 | 1.775 | -4.900 | 7.237 | 7.174 | 0.8940 | 0.5383 |
| 11949 | Da | *Myrmecobius fasciatus* | 6.155 | 2.398 | -5.196 | 7.272 | 8.735 | 0.8606 | 0.5326 |
| 01610 | Da | *Sminthopsis leadbeateri* | 2.708 | 1.609 | -4.514 | 7.374 | 8.269 | 0.8525 | 0.5374 |

Abbreviations: Af, Afrosoricida; Ar, Artiodactyla: Ca, Carnivora; Ce, Cetacea; Ch, Chiroptera; Ci, Cingulata; Da, Dasyuromorphia; Di, Diprotodontia; Eu, Eulipotyphla; La, Lagomorpha; Ma, Macroscelidea; Pe, Perissodactyla; Pi, Pilosa; Ro, Rodentia; Pr, Primates; Si, Sirenia; Tu, Tubulidentata. Currently, Cetacea is included in Cetartiodactyla, because cetaceans are nested within the paraphyletic Artiodactyla; but in this paper we continue to use the old names just to emphasize the uniqueness of whales. A total of 744 complete mammalian mitochondrial protein amino acid sequences were collected from the NCBI database (<http://www.ncbi.nlm.nih.gov/genomes/>); and 374 data sets of basal metabolic rate (*BMR*), maximum lifespan (*MLS*), and body masses (*M*), from the AnAge database (The Animal Ageing & Longevity Database; http: //genomics. senescence. info /species/; Secor 2003). We analyzed 72 mammalian species with species-to-species coincidence in these 2 databases. The *MLS* data on 6 animals (*Artibeus jamaicensis*, *Elephas maximus*, *Hemiechinus auritus*, *Homo sapiens*, *Phoca* *vitulina*, *Sus scrofa*, *Tamandua tetradactyla*) were given by Moosmann and Behl (2008). *M*, *MLS,* and *mtMR* denote the body mass (g), the maximum lifespan (yrs), and the mt metabolic rate (a. u.), respectively. *SC*, *TC*, *CC*, and *HYD* denote the Ser, Thr, and Cys contents and hydrophobicity of mtDNA-encoded membrane proteins, respectively (the estimation method of these variable values was documented in Materials and Methods 4.3). Here, we used the amino-acid sequences of 6 large subunits (ND4, ND5, ND2, CO1, CO3, and CYTB) of complexes I, IV, and III.
