# Supplementary material for: Mitochondrial determinants of mammalian longevity
Source: Open Biol. 2017 Oct 25;7(10):170083. doi: 10.1098/rsob.170083 (PMC5666079; doi:10.1098/rsob.170083)
Supplement: Table S2. Basic data for the stratified analysis found in Figs. 4-5 [file rsob170083supp2.docx]

**Table S2**. **Basic data for the stratified analysis shown in Figs. 3B and 4**

|  | NCBI | Order | Species | ln(*MLS*) | ln(*mtMR*) | *W* | *SC*(%) | *TC*(%) | *CC*(%) | *HYD* |
| --- | --- | --- | --- | --- | --- | --- | --- | --- | --- | --- |
| 1 | 06853 | Ar | *Bos taurus* | 3.401 | -5.350 | 0.096 | 7.175 | 7.982 | 0.5381 | 0.5452 |
| 1 | 09849 | Ar | *Camelus dromedarius* | 3.346 | -5.277 | 0.115 | 6.928 | 8.058 | 0.6839 | 0.5422 |
| 1 | 07704 | Ar | *Cervus elaphus* | 3.450 | -5.251 | 0.047 | 7.154 | 7.692 | 0.5388 | 0.5514 |
| 1 | 07703 | Ar | *Rangifer tarandus* | 3.077 | -5.010 | 0.040 | 7.147 | 7.772 | 0.6552 | 0.5534 |
| 1 | 00845 | Ar | *Sus scrofa* | 3.045 | -5.242 | 0.156 | 7.195 | 7.981 | 0.6348 | 0.5412 |
| 1 | 15889 | Ar | *Ovis canadensis* | 3.025 | -4.918 | 0.094 | 6.904 | 8.474 | 0.5926 | 0.5485 |
| 1 | 15247 | Ar | *Odocoileus virginianus* | 3.073 | -4.962 | 0.045 | 7.179 | 7.924 | 0.6554 | 0.5477 |
| 1 | 12103 | Ar | *Lama glama* | 3.450 | -4.916 | 0.119 | 7.318 | 8.679 | 0.6048 | 0.5380 |
| 1 | 12102 | Ar | *Pecari tajacu* | 3.364 | -5.052 | 0.145 | 7.141 | 7.941 | 0.6519 | 0.5415 |
| 1 | 01788 | Pe | *Equus asinus* | 3.850 | -5.094 | 0.550 | 7.701 | 7.731 | 0.5672 | 0.500 |
| 1 | 05212 | Ca | *Acinonyx jubatus* | 3.020 | -5.026 | 0.118 | 7.083 | 8.363 | 0.5655 | 0.5376 |
| 1 | 09691 | Ca | *Ailurus fulgens* | 2.944 | -5.032 | 0.093 | 7.117 | 8.074 | 0.6280 | 0.5577 |
| 1 | 08093 | Ca | *Canis latrans* | 3.082 | -4.951 | 0.120 | 7.128 | 7.697 | 0.6588 | 0.5502 |
| 1 | 09692 | Ca | *Enhydra lutris* | 3.296 | -4.665 | 0.036 | 7.331 | 8.277 | 0.5912 | 0.5402 |
| 1 | 09685 | Ca | *Gulo gulo* | 2.970 | -4.863 | 0.054 | 7.253 | 8.026 | 0.5945 | 0.5429 |
| 1 | 06835 | Ca | *Herpestes javanicus* | 2.809 | -4.664 | 0.198 | 7.469 | 7.772 | 0.5745 | 0.5397 |
| 1 | 09970 | Ca | *Melursus ursinus* | 3.506 | -5.307 | 0.102 | 7.171 | 7.861 | 0.6601 | 0.5428 |
| 1 | 01325 | Ca | *Phoca vitulina* | 3.689 | -5.212 | 0.061 | 7.394 | 8.110 | 0.6212 | 0.5412 |
| 1 | 09126 | Ca | *Procyon lotor* | 3.045 | -4.898 | 0.032 | 7.225 | 8.128 | 0.7225 | 0.5473 |
| 1 | 08434 | Ca | *Vulpes vulpes* | 3.059 | -4.696 | 0.099 | 7.482 | 7.481 | 0.6335 | 0.5432 |
| 1 | 14456 | Ca | *Lynx rufus* | 3.475 | -4.758 | 0.158 | 6.939 | 7.981 | 0.5956 | 0.5401 |
| 1 | 01325 | Ca | *Phoca vitulina* | 3.863 | -5.212 | 0.061 | 7.394 | 8.110 | 0.6261 | 0.5412 |
| 1 | 10497 | Ca | *Spilogale putorius* | 2.351 | -4.750 | 0.066 | 7.540 | 7.959 | 0.6882 | 0.5398 |
| 1 | 11358 | Ca | *Lutra lutra* | 2.901 | -4.661 | 0.038 | 7.101 | 8.509 | 0.5869 | 0.5366 |
| 1 | 10642 | Ca | *Panthera tigris* | 3.270 | -5.036 | 0.142 | 7.102 | 8.271 | 0.6892 | 0.5420 |
| 1 | 09126 | Ca | *Procyon lotor* | 3.045 | -4.898 | 0.032 | 7.225 | 8.128 | 0.7225 | 0.5473 |
| 1 | 02009 | Ch | *Artibeus jamaicensis* | 2.302 | -4.402 | 0.740 | 7.217 | 8.577 | 0.5620 | 0.5507 |
| 1 | 02619 | Ch | *Pteropus scapulatus* | 2.760 | -4.600 | 0.429 | 7.538 | 7.718 | 0.5384 | 0.5436 |
| 1 | 05434 | Ch | *Pteropus pumilus* | 2.845 | -4.677 | 0.695 | 7.424 | 8.176 | 0.5711 | 0.5525 |
| 1 | 07393 | Ch | *Rousettus egyptiacus* | 3.131 | -4.540 | 0.459 | 7.564 | 7.862 | 0.5360 | 0.5451 |
| 1 | 02808 | Eu | *Echinosorex gymnura* | 1.609 | -4.817 | 0.766 | 7.642 | 7.434 | 0.7731 | 0.5582 |
| 1 | 02080 | Eu | *Erinaceus europaeus* | 2.460 | -4.703 | 0.341 | 7.824 | 6.436 | 0.7972 | 0.5567 |
| 1 | 05033 | Eu | *Hemiechinus auritus* | 1.946 | -4.836 | 0.419 | 7.098 | 7.158 | 0.8613 | 0.5529 |
| 2 | 00884 | Ro | *Cavia porcellus* | 2.485 | -4.436 | 1.220 | 7.727 | 7.848 | 0.7244 | 0.5474 |
| 2 | 05314 | Ro | *Jaculus jaculus* | 1.988 | -4.290 | 1.136 | 8.483 | 7.407 | 0.6571 | 0.5472 |
| 2 | 05089 | Ro | *Mus musculus* | 1.386 | -4.247 | 0.451 | 7.577 | 7.597 | 0.6888 | 0.5515 |
| 2 | 01892 | Ro | *Myoxus glis* | 2.163 | -4.361 | 1.582 | 8.260 | 7.592 | 0.6984 | 0.5481 |
| 2 | 005315 | Ro | *Nannospalax ehrenbergi* | 2.708 | -4.408 | 0.915 | 7.657 | 8.323 | 0.7869 | 0.5439 |
| 2 | 001665 | Ro | *Rattus norvegicus* | 1.609 | -4.377 | 0.276 | 7.713 | 8.105 | 0.7532 | 0.5479 |
| 2 | 015112 | Ro | *Heterocephalus glaber* | 3.343 | -4.408 | 1.032 | 8.544 | 7.641 | 0.6919 | 0.5308 |
| 2 | 014858 | Ro | *Rattus lutreolus* | 1.482 | -4.430 | 0.209 | 7.776 | 8.137 | 0.7233 | 0.5440 |
| 2 | 014867 | Ro | *Rattus fuscipes* | 1.668 | -4.409 | 0.184 | 7.880 | 7.789 | 0.7218 | 0.5439 |
| 2 | 013276 | Ro | *Mesocricetus auratus* | 1.361 | -4.335 | 0.627 | 7.888 | 7.828 | 0.8398 | 0.5465 |
| 2 | 001913 | La | *Oryctolagus cuniculus* | 2.893 | -4.393 | 3.467 | 7.411 | 7.770 | 0.5977 | 0.5518 |
| 3 | 06901 | Pr | *Colobus guereza* | 3.555 | -5.252 | 0.585 | 6.889 | 9.403 | 0.6505 | 0.5365 |
| 3 | 01807 | Pr | *Homo sapiens* | 4.605 | -5.428 | 0.924 | 7.043 | 9.183 | 0.5349 | 0.5359 |
| 3 | 01992 | Pr | *Papio hamadryas* | 3.624 | -5.433 | 0.473 | 7.056 | 10.15 | 0.5148 | 0.5246 |
| 3 | 02765 | Pr | *Nycticebus coucang* | 3.250 | -5.259 | 0.683 | 7.766 | 8.426 | 0.6597 | 0.5394 |
| 3 | 12766 | Pr | *Eulemur fulvus* | 3.570 | -5.367 | 1.989 | 7.449 | 8.277 | 0.7064 | 0.5407 |
| 3 | 12775 | Pr | *Saimiri sciureus* | 3.408 | -4.763 | 0.957 | 7.197 | 8.951 | 0.6048 | 0.5418 |
| 3 | 12774 | Pr | *Tarsius syrichta* | 2.773 | -4.897 | 1.989 | 7.310 | 8.079 | 0.6511 | 0.5475 |
| 3 | 12764 | Pr | *Perodicticus potto* | 3.288 | -5289 | 0.865 | 7.973 | 8.940 | 0.6041 | 0.5262 |
| 3 | 12763 | Pr | *Loris tardigradus* | 2.960 | -4.986 | 0.683 | 8.142 | 8.535 | 0.5751 | 0.5302 |
| 3 | 12762 | Pr | *Otolemur crassicaudatus* | 3.122 | -5.097 | 0.860 | 7.834 | 8.7724 | 0.538 | 0.5288 |
| 4 | 005268 | Ce | *Balaena mysticetus* | 5.352 | -11.236 | 0.5714 | 6.732 | 8.758 | 0.6255 | 0.5423 |
| 4 | 005271 | Ce | *Bala. acutorostrata* | 3.912 | -10.252 | 0.2556 | 6.617 | 8.554 | 0.6259 | 0.5463 |
| 4 | 006929 | Ce | *Balaenoptera borealis* | 4.304 | -10.624 | 0.1203 | 6.762 | 8.520 | 0.5958 | 0.5448 |
| 4 | 007938 | Ce | *Balaenoptera edeni* | 4.277 | -10.540 | 0.1203 | 6.736 | 8.902 | 0.6232 | 0.5404 |
| 4 | 001601 | Ce | *Balaenoptera musculus* | 4.700 | -11.353 | 0.1805 | 6.853 | 8.573 | 0.6526 | 0.5429 |
| 4 | 001321 | Ce | *Balaenoptera physalus* | 4.736 | -11.100 | 0.1805 | 6.814 | 8.428 | 0.5977 | 0.5462 |
| 4 | 005274 | Ce | *Berardius bairdii* | 4.431 | -10.411 | 0.9774 | 6.924 | 9.034 | 0.5944 | 0.5454 |
| 4 | 005270 | Ce | *Macropus robustus* | 4.344 | -10.762 | 0.3609 | 6.667 | 8.720 | 0.6250 | 0.5433 |
| 4 | 005276 | Ce | *Inia geoffrensis* | 3.443 | -8.545 | 3.4734 | 6.625 | 9.642 | 0.5915 | 0.5355 |
| 4 | 005272 | Ce | *Kogia breviceps* | 2.833 | -9.160 | 0.9925 | 6.830 | 9.424 | 0.5667 | 0.5347 |
| 4 | 007629 | Ce | *Lipotes vexillifer* | 3.178 | -8.542 | 2.6917 | 6.909 | 9.300 | 0.6495 | 0.5339 |
| 4 | 006927 | Ce | *Megaptera novaeangliae* | 4.554 | -10.778 | 0.1805 | 6.744 | 8.903 | 0.6211 | 0.5408 |
| 4 | 005279 | Ce | *Monodon monoceros* | 3.912 | -9.571 | 7.143 | 7.143 | 9.056 | 0.5380 | 0.5412 |
| 4 | 005280 | Ce | *Phocoena phocoena* | 3.016 | -8.366 | 2.2105 | 6.784 | 7.747 | 0.5951 | 0.5517 |
| 4 | 002503 | Ce | *catodon* | 4.344 | -10.911 | 0.8421 | 6.607 | 9.970 | 0.6306 | 0.5341 |
| 5 | 02631 | Af | *Echinops telfairi* | 2.944 | -4.968 | 1.0510 | 8.072 | 7.162 | 0.6163 | 0.5616 |
| 5 | 06924 | Pi | *Cyclopes didactylus* | 3.332 | -5.281 | 0.9287 | 7.212 | 9.525 | 0.6911 | 0.5352 |
| 5 | 04032 | Pi | *Tamandua tetradactyl* | 2.197 | -5.744 | 0.9514 | 6.864 | 7.802 | 0.7040 | 0.5417 |
| 5 | 04026 | Ma | *Macro. proboscideus* | 2.163 | -4.638 | 0.9687 | 7.848 | 8.175 | 0.7432 | 0.5409 |
| 5 | 02078 | Tu | *Orycteropus afer* | 3.394 | -6.137 | 1.3106 | 7.769 | 7.065 | 0.8329 | 0.5570 |
| 5 | 10302 | Si | *Trichechus manatus* | 4.025 | -6.705 | 1.1088 | 7.720 | 7.780 | 0.7481 | 0.5503 |
| 5 | 05129 | Ci | *Elephas maximus* | 4.382 | -6.073 | 0.6309 | 7.190 | 9.010 | 0.6265 | 0.5384 |
| 5 | 01821 | Ci | *Dasypus novemcinctus* | 3.105 | -5.906 | 1.0907 | 7.141 | 8.301 | 0.6843 | 0.5475 |
| 5 | 07630 | Di | *Potorous maculatus* | 2.674 | -5.466 | 0.6700 | 6.937 | 8.083 | 0.9406 | 0.5545 |
| 5 | 06519 | Di | *Pseudo peregrinus* | 2.313 | -5.230 | 1.0690 | 7.266 | 7.899 | 0.8743 | 0.5474 |
| 5 | 03039 | Di | *Trichosurus vulpecula* | 2.766 | -5.536 | 1.1360 | 7.442 | 8.105 | 0.8135 | 0.5416 |
| 5 | 01794 | Di | *Macropus robustus* | 3.091 | -5.751 | 1.1360 | 7.160 | 7.751 | 0.8580 | 0.5509 |
| 5 | 08134 | Di | *Petaurus breviceps* | 2.879 | -4.897 | 0.8970 | 7.469 | 7.828 | 0.8398 | 0.5477 |
| 5 | 08133 | Di | *Phascolarctos cinereus* | 3.096 | -5.784 | 1.5240 | 7.268 | 9.439 | 0.9650 | 0.5354 |
| 5 | 06523 | Da | *Phascogale tapoatafa* | 1.775 | -5.056 | 0.7010 | 6.700 | 7.672 | 0.9663 | 0.5543 |
| 5 | 11949 | Da | *Myrmecobius fasciatus* | 2.398 | -5.422 | 1.0010 | 7.242 | 8.277 | 1.005 | 0.5444 |
| 5 | 01610 | Da | *Sminthopsis leadbeateri* | 1.609 | -4.580 | 0.8280 | 7.300 | 7.740 | 0.9088 | 0.5519 |

Abbreviations: Af, Afrosoricida; Ar, Artiodactyla: Ca, Carnivora; Ce, Cetacea; Ch, Chiroptera; Ci, Cingulata; Da, Dasyuromorphia; Di, Diprotodontia; Eu, Eulipotyphla; La, Lagomorpha; Ma, Macroscelidea; Pe, Perissodactyla; Pi, Pilosa; Ro, Rodentia; Pr, Primates; Si, Sirenia; Tu, Tubulidentata. The first column denotes the 5 animal groups (N=1-5). *MLS,* *mtMR* and *W* denote the maximum lifespan (yrs), the mt metabolic rate (a. u.) and the phylogenetic weight, respectively. *SC*, *TC*, *CC*, and *HYD* refer to the Ser, Thr, and Cys contents and hydrophobicity of mtDNA-encoded membrane proteins, respectively. The values of variables (*MLS*, *W*, *mtMR*, *SC*, *TC*, *CC*, and *HYD*) are listed. Here, we used the complete amino-acid sequences of all 13 proteins. The coefficients (*Ai*, *i*=0-4) in the 5 animal groups are as follow:

1. Laurasiatheria: A_1_=1.0955, A_2_=-0.2298, A_3_=-9.8398, A_4_=-1.4322, A_0_=-1.7201

2. Rodents: A_1_=1.0289, A_2_=2.3849, A_3_=-4.1904, A_4_=-42.0586, A_0_=-3.2168

3. Primates: A_1_=-0.4466, A_2_=0.6752, A_3_=-1.5907, A_4_=-1.6315, A_0_=-2.5397

4. Cetaceans: A_1_=-0.0429, A_2_=-0.1714, A_3_=0.2435, A_4_=4.0870, A_0_=-6.9390

5. Others: A_1_=0.9514, A_2_=0.2522, A_3_=,-1.2723 A_4_=-16.1261, A_0_=-2.0287
