# Supplementary material for: Mitochondrial determinants of mammalian longevity
Source: Open Biol. 2017 Oct 25;7(10):170083. doi: 10.1098/rsob.170083 (PMC5666079; doi:10.1098/rsob.170083)
Supplement: Figure S1. Rodent phylogeny. [file rsob170083supp3.docx]

**Figure S1.** Rodent phylogeny. Rodents were separated into 2 groups of rats/mice, one with a small *MLS* and the other with a large *MLS*.
