# Supplementary material for: Mitochondrial determinants of mammalian longevity
Source: Open Biol. 2017 Oct 25;7(10):170083. doi: 10.1098/rsob.170083 (PMC5666079; doi:10.1098/rsob.170083)
Supplement: Figure S2. Phylogenetic analysis of the rodent lineage. [file rsob170083supp4.docx]

**Figure S2.** Phylogenetic analysis of the rodent lineage. *MLS* and *mtMR* denote maximum life span and metabolic rate per mitochondrion in an arbitrary unit (a. u.), respectively. The optimization procedure given by adjusting α in equation 2 provided mtMR≈constant at *α* = 8.0. The variable values used in this analysis are listed in Table S2.
